# Preconception Care: Assessing Knowledge, Attitudes, and Practices among Physicians at a Tertiary Hospital in Ethiopia

**DOI:** 10.1055/a-2667-6662

**Published:** 2025-08-12

**Authors:** Habtamu Dagnew Demsew, Atirsaw Ebabey, Winta Tsehaye, Nigat Amsalu Addis, Adane Nigusie, Nurhussien Riskey Arefayne, Demelash Gedefaye Anteneh, Belete Muluadam Admassie

**Affiliations:** 1 Department of Gynecology and Obstetrics, College of Medicine and Health Sciences, University of Gondar, Gondar, Ethiopia; 2 Department of Public Health and Health Promotion, College of Medicine and Health Sciences, University of Gondar, Gondar, Ethiopia; 3 Department of Anesthesia, College of Medicine and Health Sciences, University of Gondar, Gondar, Ethiopia; 4 Department of Anesthesia, College of Medicine and Health Sciences, Bahir Dar University, Bahir Dar, Ethiopia

**Keywords:** attitude, knowledge, practice, preconception care, physician

## Abstract

**Background:**

Preconception care (PCC) involves interventions before conception to prevent adverse pregnancy outcomes, yet it remains underutilized in many regions. Its availability influenced by both women's awareness and HCPs' expertise. This study aimed to assess physician's knowledge, attitudes, and practices regarding PCC.

**Objective:**

To assess physicians knowledge, attitudes, and practices related to PCC and associated factors.

**Methods:**

A cross-sectional study was conducted from March 20 to May 30, 2023, on 251 physicians. Data were collected using a pretested, semi-structured questionnaire and analyzed using Stata version 14. Bivariate and multivariable logistic regression were used to identify factors associated with PCC knowledge, attitudes, and practices.

**Results:**

From 251 physicians, 133 (52.99%) had strong PCC practices, 180 (71.71%) had good knowledge, and 143 (56.97%) demonstrated positive attitudes. Significant factors influencing knowledge included working department (adjusted odds ratio [AOR] = 3.02) and reading PCC guidelines (AOR = 1.82). Strong PCC practices were linked to working department (AOR = 2.74), reading about PCC (AOR = 2.86), and perceptions of who should provide PCC (AOR = 2.21).

**Conclusion and Recommendation:**

Physicians' expertise in PCC is enhanced by reading guidelines and working in obstetrics and gynecology. Regular review of PCC resources is recommended to improve knowledge and practices.

## Background

Preconception care (PCC) is a set of interventions designed to identify and manage biological, behavioral, and social factors that may affect a woman's health and pregnancy outcomes. In 2017, an estimated 295,000 maternal deaths occurred globally, with sub-Saharan Africa and South Asia accounting for 86% of these fatalities. 1

PCC aims to optimize women's health, behaviors, and knowledge before conception through risk assessment, health promotion, and medical and psychosocial interventions. Initially focused on women with previous adverse pregnancy outcomes, PCC is now recommended for all women to reduce the risk of complications. 2 By addressing key health factors before pregnancy, PCC improves maternal and neonatal outcomes, emphasizing the importance of early intervention and comprehensive care. 3

PCC includes a comprehensive range of interventions to optimize maternal health before pregnancy. Essential components include nutritional assessment, smoking cessation, genetic screening, infertility management, and environmental health considerations. PCC also addresses interpersonal violence, sexually transmitted infection treatment, human immunodeficiency virus (HIV) prevention, mental health support, substance use prevention, vaccination, and efforts to prevent female genital mutilation. By integrating these interventions, PCC reduces pregnancy-related risks and improves maternal and neonatal health outcomes. 4

Adverse reproductive outcomes resulting from abnormal organ development due to drug use, alcohol consumption, and poor nutrition cannot be effectively addressed by traditional early prenatal visits. 5 Research from Ethiopia indicates that the average timing of the first antenatal care (ANC) visit is 15.9 weeks, which may be too late to prevent complications linked to early fetal development. 6 According to the 2016 Ethiopian Demographic Health Survey, only 20% of women received their first ANC visit during the first trimester. 7 Ethiopia's current maternal health strategy primarily focuses on child health, ANC, intrapartum care, and postnatal care. However, these interventions often begin too late after conception and pregnancy awareness failing to address critical risk factors that influence early fetal development. This underscores the need for stronger PCC initiatives to optimize maternal health before pregnancy, ultimately improving pregnancy and neonatal outcomes.

## Statement of Problem

Data from the Centers for Disease Control and Prevention (CDC) Pregnancy Risk Assessment Monitoring System highlight concerning preconception health behaviors: 23.2% of women smoked, 50.1% consumed alcohol, and only 35.1% took multivitamins regularly in the 3 months before pregnancy. Additionally, 10% continued drinking alcohol, and 11% continued smoking after conception. Recognizing the impact of these behaviors on maternal and neonatal health, health experts and policymakers emphasize the importance of PCC. International organizations advocate for PCC as a critical strategy to improve pregnancy outcomes and reduce preventable risks. 1 5 8 9 10 11 Despite widespread recognition of the importance of PCC by health experts and international organizations, many countries, including Ethiopia, have not yet translated this awareness into national policy. 12 As a result, the implementation of PCC remains inconsistent across clinical settings. Numerous studies indicate that health care practitioners are often unclear about who is responsible for delivering PCC, contributing to fragmented and uneven access to these critical services. This highlights the urgent need for clear policies and guidelines to ensure comprehensive and equitable delivery of PCC. 13

A systematic review highlights that several provider attributes, such as attitudes and communication with other clinicians, can significantly influence the utilization of PCC. 14 This suggests that health care professionals (HCPs) play a crucial role in shaping couples' decisions to seek PCC. However, despite this potential, HCPs typically provide PCC infrequently and mainly in an opportunistic manner, rather than as part of regular, systematic care. This inconsistency underscores the need for more structured and consistent delivery of PCC by health care providers. 15 To create an effective national PCC program, it is crucial to understand health care providers' current practices, attitudes, and perceptions of obstacles to successful implementation. 16 This insight will help identify challenges, inform policy, and guide the development of strategies to ensure consistent and effective delivery of PCC across clinical settings.

A study conducted on physicians (residents) revealed a significant gap in the delivery of PCC, despite positive knowledge and attitudes about the subject. However, the reasons for the poor practice of PCC were not identified. 7

HCPs' attitudes, expertise, and ability to educate patients are crucial in the use of PCC services. Despite positive attitudes, a 1991 study of internal medicine and family practice residents at a public hospital found that both groups exhibited poor decision-making skills and limited knowledge about PCC, highlighting the need for enhanced training to improve effective PCC delivery. 17

A cross-sectional study in Iran found that 70% of doctors practice PCC, with 63.6% demonstrating moderate expertise, and all having a positive attitude. 18 In contrast, a survey of obstetrician–gynecologists among American College of Obstetricians and Gynecologists (ACOG) members showed that 87% considered PCC necessary, and 94% advised it to women planning pregnancy, although only a few pregnant patients sought PCC, suggesting a gap between awareness and usage. 19 A study in India revealed that while 92.07% of doctors were aware of folic acid, only 47.52% were knowledgeable about preconception administration, indicating significant gaps in PCC-related knowledge. 20

In Canada, a survey found that half of the doctors were aware of the correct dosage of folic acid, but more than two-thirds were unclear about the latest guidelines, underscoring the need for continuous updates in training and awareness. 21 A systematic review in Africa found that only 18.72% of pregnant women used PCC services, with knowledge and preexisting conditions being strong predictors of PCC utilization. 22 In Egypt, 22% of primary HCPs had strong PCC knowledge, whereas 48.5% had a positive attitude toward PCC. 23 Studies in Ethiopia revealed that although health care workers demonstrated strong knowledge and favorable attitudes, practical implementation was suboptimal. In one study, only 19.2% of doctors exhibited good practice despite strong knowledge, 4 whereas another found that 30.9% of consultants had poor practice despite 85.7% showing strong knowledge and 83.1% favorable attitudes. 24 Education, professional experience, and exposure to PCC guidelines are strong predictors of knowledge. In Iran, HCPs with more education demonstrated better knowledge of PCC. 18 Similarly, studies in Canada and Ethiopia found that years of professional practice and exposure to PCC guidelines correlated with better knowledge. 21 25 Training in PCC, such as through education on HIV testing and human immune virus management, has also been shown to positively impact health care providers' knowledge of PCC. 26

Health professionals with strong knowledge of PCC are more likely to have positive attitudes. Studies from the Netherlands and Nigeria show that many health care providers believe PCC should be provided across all levels of health care, with some expressing the belief that all HCPs have a role in delivering PCC services. 27 28 In Ethiopia, health care providers who had read PCC service guidelines or had prior training were more likely to have a favorable attitude. 29

HCPs who possess a positive attitude and strong knowledge of PCC are more likely to implement it in practice. However, the lack of practice among health care providers remains a challenge. A systematic review identified that those with a negative attitude or inadequate knowledge, particularly outside obstetrics and gynecology, were less likely to provide PCC. 13 Studies in Ethiopia show that obstetric care providers trained in reproductive life plan screening were more likely to practice PCC effectively, although no significant correlation between knowledge and practice was observed. 30 31

## Significance of the Study

Several well-known international organizations, such as the World Health Organization (WHO), the ACOG, and the CDC, recommend PCC for all women and men of childbearing age due to its potential to improve pregnancy outcomes. 9 10 32

However, in Ethiopia, HCPs generally have a limited understanding and less favorable attitudes toward PCC compared with those in economically developed nations. 17 18 19 The knowledge, attitudes, and practices of doctors, particularly interns, residents, and consultants, significantly influence the use of PCC. Despite its importance, there is a lack of research focusing on physicians' knowledge, attitudes, and practices regarding PCC in Ethiopia, with limited studies involving interns who are often the first-line medical professionals and have frequent patient interactions.

This study aims to evaluate the knowledge, attitudes, and practices related to PCC among physicians at the University of Gondar Comprehensive Specialized Hospital (UOGCSH). The findings will provide valuable insights for the obstetrics and gynecology department to make necessary modifications to better meet the reproductive health needs of women. Additionally, the study will assess the quality of PCC services provided at a tertiary hospital like UOGCSH, addressing the unique needs of women with various medical conditions. Ultimately, the results will serve as a baseline for future research and the development of effective PCC programs in Ethiopia.

## Materials and Methods

### Study Period and Setting

**Fig. 1:**
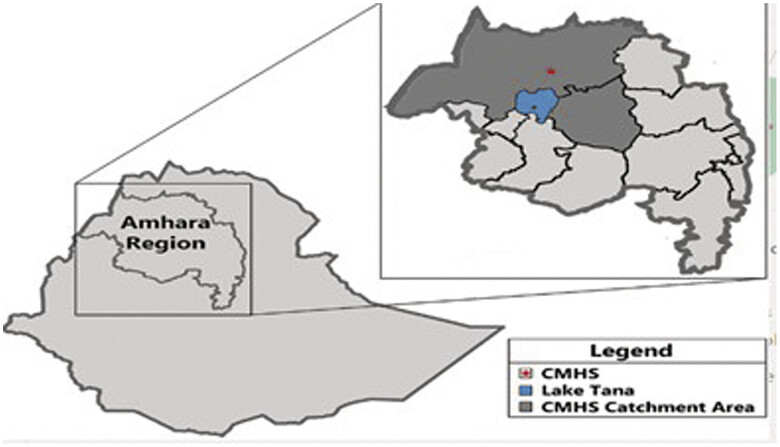
Graphic maps of UoGCSH examined for PCC practice and utilization. PCC, preconception care; UoGCSH, University of Gondar Comprehensive Specialized Hospital.

This study was conducted at the UOGCSH, one of Ethiopia's leading teaching hospitals, located in Gondar city, approximately 743 km from the capital, Addis Ababa ( Fig. 1 . The hospital serves over 7 million patients and has more than 700 beds, 177 of which are dedicated to obstetrics and gynecology services. From May 1 to May 30, 2023, we focused on three key departments where the majority of our clients, particularly those at risk of unfavorable pregnancy outcomes, seek preconception counseling. According to the hospital data, the UOGCSH manages approximately over 10,000 deliveries annually. Trainees, including interns and residents, actively participate in PCC provision, especially in the Obstetrics and Gynecology Department, where PCC elements such as family planning, risk assessment, and nutritional counseling are routinely provided. This selection was made to gather comprehensive data on the knowledge, attitudes, and practices related to PCC.

### Study Design

This is an institution-based, cross-sectional study.

### Source Population

The source population for this study was all physicians working in UOGCSH during the study period.

### Study Populations

All interns and residents working at department of internal medicine, psychiatry, and gynecology and obstetrics during the study period

### Inclusion and Exclusion Criteria

All eligible physicians, including interns and residents from the Internal Medicine, Psychiatry, and Obstetrics and Gynecology, participated in the survey. Participants who did not volunteer to take part in the study or those who were on annual leave during the study period were excluded from the research. Consultants were excluded due to their limited number and unavailability during the study period. This approach ensured that the sample accurately represented the HCPs actively involved in providing care in the Obstetrics and Gynecology Department.

### Sample Size Determination

Since population size is small (254) and manageable, it is reasonable to include all of the study population

### Variables of Study

#### Dependent Variables

Knowledge, attitude, and practice.

#### Independent Variables

Sociodemographic factors: age, gender, religion, years of service, marital status, nationality.

Behavioral and occupational factors: training on PCC, ever-read PCC guideline, types of institution they work while they are general practitioner, department, the perceived expectation on who should give PCC service, opinions on PCC provision site.

## Operational Definition

### Preconception Care

PCC is the provision of biomedical, behavioral, and social health interventions to women and couples before conception occurs.

### Physicians

#### Knowledge of Preconception Care

In this study, knowledge of PCC was evaluated through 24 questions, with responses based on a 5-point Likert scale ranging from “strongly disagree” to “strongly agree.” Participants who answered 60% or more of the questions correctly were classified as having “good PCC knowledge,” whereas those who scored below 60% were categorized as having “low/poor PCC knowledge.” This approach provided a clear measure of HCPs' understanding of PCC. 24

#### Attitude of Preconception Care

In this study, attitudes refer to physicians' feelings and beliefs regarding PCC. These were measured using 12 questions with responses on a 5-point Likert scale, ranging from “strongly disagree” to “strongly agree.” Physicians who answered 60% or more of the questions correctly were categorized as having a “positive PCC attitude,” whereas those who scored below 60% were classified as having a “negative/unfavorable PCC attitude.” This measurement helped assess the overall perception of PCC among health care providers. 24

#### Practice of Preconception Care

In this study, practice refers to the implementation of PCC. It was measured using nine questions, with responses on a 5-point Likert scale ranging from “never” to “always,” assessing the frequency with which health care providers implement various components of PCC. Those who scored 60% or more of the total composite score were classified as having “good PCC practice,” while those who scored below 60% were categorized as having “low/poor PCC practice.” This evaluation provided insight into the actual delivery of PCC by health care providers. 24

## Data Collection Procedure

Data for this study were collected using a self-administered, pretested questionnaire, which was modified based on CDC and WHO guidelines. The reliability of the instrument was assessed using Cronbach's α, yielding results of 0.79 for knowledge and 0.774 for practice, indicating good internal consistency. The content validity index of the questionnaire was 92.4%, ensuring that the instrument accurately measured the intended constructs. 31 The questionnaire used in this study was developed from a previously validated instrument. Physicians' consent to participate was obtained before data collection. The questionnaire was then converted into an online data collection application called Kobo Collect, and data were gathered using Android phones. This digital approach ensured efficient and organized data collection for the study.

### Data Quality Control

A structured, pretested, English-language self-administered questionnaire was used to collect data for the study. The data collectors received training on the study's objectives, the content of the questions, and proper data collection procedures. After data collection, the lead investigator reviewed the gathered data to ensure accuracy, consistency, and completeness. The final data were then presented in tables and graphs for analysis.

## Data Processing and Analysis

The data collected using the Kobo data collection tool were exported to Stata version 14 for analysis. To explore the relationship between dependent and independent variables, binary logistic regression was applied. The normality of the data was assessed using the Shapiro–Wilk test. Descriptive statistics and cross-tabulation were performed, and the findings were presented through text, tables, and graphs.

Factors associated with the dependent variables were identified using binary logistic regression. Multivariable logistic regression analysis was conducted on variables with a *p* -value less than 0.25 in the bivariable analysis. The crude odds ratio (COR) and adjusted odds ratio (AOR), along with their 95% confidence intervals, were calculated to assess the strength of association between the independent and dependent variables. In the multivariable analysis, variables with a *p* -value less than 0.05 were considered statistically significant.

## Results

### Sociodemographic Characteristics of the Respondents

A total of 251 individuals participated in the study. The majority of the respondents were male, comprising 203 participants (80.88%). The median age of participants was 28 years, with an interquartile range of 26 to 30 years ( Table 1 ).

**Table 1:** Sociodemographic characteristics of physicians working at the University of Gondar Comprehensive Specialized Hospital

Variables	Category	Frequency	%
**Age**	20–24	15	5.98
	25–29	160	63.75
	30–34	67	26.69
	≥35	9	3.59
**Sex**	Female	48	19.12
	Male	203	80.88
**Marital** s **tatus**	Married	73	29.08
	Single	178	70.92

### Professional-Related Characteristics of the Respondents

Residents accounted for the majority of the respondents, with 143 participants (56.97%), and 40.56% of them were first-year residents. The majority of respondents, 219 (87.25%), had less than 5 years of experience. Additionally, 134 (53%) were employed in the Obstetrics and Gynecology Department.

Regarding attitudes toward PCC, nearly two-thirds (161, or 64.14%) of the physicians agreed that PCC should be offered by all HCPs in addition to other treatments. However, almost half of the respondents (115, or 46%) reported that they had never read any PCC guidelines ( Table 2 ).

**Table 2:** Professional-related characteristics

Variables	Category	Frequency	%
**Level of profession**	Intern	108	43.03
	Resident	143	56.97
**Year of residency**	First	58	40.56
	Second	38	26.57
	Third	34	23.78
	Fourth	13	9.09
**Type of institution working as GP**	Primary hospital	53	37.06
	General hospital	21	14.69
	Referral hospital	68	47.55
	Other	1	0.70
**Current department**	Internal medicine	108	43.03
	Gynecology and obstetrics	134	53.39
	Psychiatry	9	3.59
**Year** s **of** e **xperience**	<5	210	83.67
	> or = 5	41	16.33
P **rior training on PCC education and counseling**	Yes	6	3.75
	No	154	96.25
**Ever** - **read PCC guideline** s **or protocol**	Yes	135	54.00
	No	115	46.00
**Do you have PCC guideline** s **specific to your working department?**	Yes	27	10.76
	No	224	89.24
**Perceived expert opinion to give PCC**	All health professionals	161	64.14
	Selected health professionals	90	35.86
**Opinion on PCC provision site**	Should be provided along with other services	142	56.5
	Should be provided in a separate clinic	109	43.43

Abbreviations: GP, general practitioners; PCC, preconception care.

### Level of Knowledge on Preconception Care

**Fig. 2:**
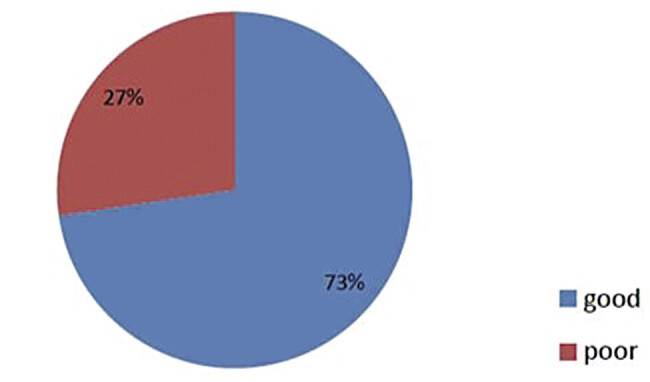
Knowledge of toward PCC among physicians. PCC, preconception care.

A total of 180 participants (72.71%) demonstrated good knowledge of PCC, with a knowledge score ranging from 56 to 110 (95% confidence interval [CI]: 43.2–53.5; Fig. 2 ).

Nearly half of the respondents (102, or 40.2%) strongly believed that PCC can reduce the incidence of birth defects. The majority (157, or 62.5%) strongly agreed that all adolescents and people of reproductive age are potential candidates for PCC. However, only 21 participants (8%) strongly believed that prenatal care should be limited to high-risk mothers ( Table 3 ).

**Table 3:** Level of knowledge on preconception care of physician working at the University of Gondar Comprehensive Specialized Hospital

Items	Level	Frequency	%
**The eligible clients for PCC include all adolescents and reproductive age individuals**	Strongly disagree Disagree Uncertain Agree Strongly agree	1 23 32 38 157	0.4 9.16 12.7 15.14 62.5
**PCC can help reduce maternal and child mortality rate** s	Strongly disagree Disagree Uncertain Agree Strongly agree	7 95 41 8 100	2.79 37.85 16.3 3.19 39.8
**PCC can lead to better pregnancy outcome** s	Strongly disagree Disagree Uncertain Agree Strongly agree	1 21 75 54 100	0.40 8.37 29.8 21.51 39.8
**Only high-risk mothers need** PCC **when planning for pregnancy**	Strongly disagree Disagree Uncertain Agree Strongly agree	21 38 21 98 73	8.37 15.14 8.37 39.04 29.08
**PCC can reduce the incidence of birth defect** s	Strongly disagree Disagree Uncertain Agree Strongly agree	17 4 43 86 101	6.77 1.59 17.1 34.26 40.2
**There is little evidence base for** PCC	Strongly agree agree Uncertain disagree Strongly disagree	17 45 77 76 36	6.77 17.93 30.68 30.28 14.34
**Family planning counseling is one component of PCC**	Strongly disagree Disagree Uncertain Agree Strongly agree	10 8 63 75 95	3.98 3.19 25.0 29.8 37.85
**Periodontal disease is a risk factor for adverse pregnancy outcomes (APO)**	Strongly disagree Disagree Uncertain Agree Strongly agree	1 21 113 81 35	0.40 8.37 45.0 32.2 13.94
**Human** papillomavirus, rubella, and varicel **la are all vaccines contraindicated during pregnancy**	Strongly disagree Disagree Uncertain Agree Strongly agree	27 85 78 9 52	10.76 33.86 31.08 3.59 20.72
**Preconception genetic counseling and screening include recommending carrier screening tests for the client with sickle cell hemoglobinopathies**	Strongly disagree Disagree Uncertain Agree Strongly agree	3 4 56 138 50	1.20 1.59 22.31 54.98 19.92
**Women need to start taking folic acid 3** **mo before pregnancy**	Strongly disagree Disagree Uncertain Agree Strongly agree	12 12 17 93 117	4.78 4.78 6.77 37.05 46.61
**The recommended test that guarantees good preconception blood sugar control for a woman with pregestational diabetes is the random blood sugar test**	Strongly agree Agree Uncertain Disagree Strongly disagree	77 94 22 36 22	30.68 37.45 8.76 14.34 8.76
**Early identification and treatment of diseases like depression and seizure disorder during the preconception period** , **reduc** ing **the occurrence of APO**	Strongly disagree Disagree Uncertain Agree Strongly agree	5 4 57 73 112	1.99 1.59 22.7 29.08 44.62
**Recommending regular exercise is an important PCC counseling point. Thus, women planning pregnancy should aim for 30 min of moderate exercise 5 d** / **wk**	Strongly disagree Disagree Uncertain Agree Strongly agree	10 12 30 131 68	3.98 4.78 11.95 52.19 27.09
**It is recommended to** a **void multiple sexual partners when plann** ing a **pregnancy**	Strongly disagree Disagree Uncertain Agree Strongly agree	90 6 26 125 4	35.86 2.39 10.36 49.80 1.59
**Screening for intimate partner violence should occur during prepregnancy counseling**	Strongly disagree Disagree Uncertain Agree Strongly agree	90 6 26 125 4	35.86 2.39 10.36 49.80 1.59
**Women planning pregnancy should be advised to delay pregnancy until reduc** ed **drug, alcohol, and tobacco use**	Strongly disagree Disagree Uncertain Agree Strongly agree	11 2 14 100 125	4.38 0.80 5.58 39.84 49.8
**We** c **heck for immunization status for women who** are **planning pregnanc** ies	Strongly disagree Disagree Uncertain Agree Strongly agree	3 11 23 115 99	1.20 4.38 9.16 45.82 39.44
**Isotretinoin,** v **alproic acid, and** w **arfarin are medications that pose teratogenic effects and requir** e **preconception modification**	Strongly disagree Disagree Uncertain Agree Strongly agree	12 2 4 91 91	4.78 0.80 1.59 36.25 36.25
**The recommended routine preconception laboratory tests include Hct, HIV,** **HBV, and RPR or VDRL tests**	Strongly disagree Disagree Uncertain Agree Strongly agree	13 4 8 93 133	5.18 1.59 3.19 37.05 52.99
**Avoidance of exposure to environmental hazards or toxins such as ionizing radiation, pesticides, lead** … **is a concern for women with established first-trimester pregnancy, not for couples planning a pregnancy**	strongly agree agree Uncertain disagree Strongly disagree	33 35 22 88 73	13.15 13.94 8.76 35.06 29.08
**Provide preconceptional counseling for men**	Strongly disagree Disagree Uncertain Agree Strongly agree	18 10 14 101 108	7.17 3.98 5.58 40.24 43.03
**A clinician attending to clients with previous cesar** e **an section** s **should advise the client to delay the next pregnancy for at least 18 mo before the next conception**	Strongly disagree Disagree Uncertain Agree Strongly agree	20 10 10 99 112	7.97 3.98 3.98 39.44 44.63
**Infertility screening and management** are **not the concern of PCC**	Strongly agree agree Uncertain Disagree Strongly disagree	15 7 31 89 109	5.98 2.79 12.35 35.46 43.43

Abbreviations: HIV, human immunodeficiency virus; PCC, preconception care.

### Level of Attitude toward Preconception Care

More than half of the participants (143, or 56.97%) had a positive opinion of PCC. The respondents' mean attitude score was 43.8, with a standard deviation ± 5.9, ranging from a minimum score of 26 to a maximum score of 56 out of 60.

Regarding the implementation of PCC, 149 respondents (59.3%) agreed that they would go above and beyond for their patients when providing PCC. Additionally, the majority (114, or 45.42%) strongly disagreed with the notion that providing PCC is outside the scope of their professional obligation and accountability ( Table 4 ).

**Table 4:** Attitude toward preconception care of physicians working at the University of Gondar Comprehensive Specialized Hospital

Items	Level	Frequency	%
**Providing PCC is not within the scope of my professional responsibility and accountability**	Strongly agree Agree Uncertain Disagree Strongly disagree	17 9 92 19 114	6.77 3.59 36.65 7.57 45.42
**Due to the presence of other competing demands, providing PCC is not the priority intervention I should provide**	Strongly agree Agree Uncertain Disagree Strongly disagree	28 8 76 30 109	11.16 3.19 30.28 11.95 43.43
**A dedicated clinic for** PCC **is a luxury service**	Strongly agree Agree Uncertain Disagree Strongly disagree	12 10 109 11 109	4.78 3.98 43.43 4.38 43.43
PCC **is a high priority in my workload**	Strongly disagree Disagree Uncertain Agree Strongly agree	24 66 29 104 28	9.56 26.29 11.55 41.43 11.16
PCC **without women asking for it is objectionable**	Strongly agree Agree Uncertain Disagree Strongly disagree	9 51 62 92 37	3.59 20.32 24.70 36.65 14.74
**Interns or residents in** obstetrics and gynecology **departments are not the best people to offer** PCC	Strongly agree Agree Uncertain Disagree Strongly disagree	12 15 24 114 86	4.78 5.98 9.56 45.42 34.26
**Interns or residents in internal medicine departments are not the best people to offer** PCC	Strongly agree Agree Uncertain Disagree Strongly disagree	22 33 36 99 61	8.76 13.15 14.34 39.44 24.30
**With** PCC **, I can do something extra for my patients**	Strongly disagree Disagree Uncertain Agree Strongly agree	3 29 20 149 50	1.20 11.55 7.97 59.36 19.92
**A hospital setting is the best place to provide** PCC	Strongly disagree Disagree Uncertain Agree Strongly agree	8 32 16 132 63	3.19 12.75 6.37 52.59 25.10
PCC **provision depends on health care** **providers willingness**	Strongly disagree Disagree Uncertain Agree Strongly agree	29 88 28 74 32	11.55 35.06 11.16 29.48 12.75
PCC **should be given for all healthy and sick individuals** , **including those presented with a critical and emergency condition**	Strongly agree Agree Uncertain Disagree Strongly disagree	50 12 43 48 39.04	19.92 4.78 17.13 19.12 39.04
**I am not comfortable offer preconceptional care**	Strongly disagree Disagree Uncertain Agree Strongly agree	17 28 7 121 78	6.77 11.16 2.79 48.21 31.08

Abbreviation: PCC, preconception care.

### Preconception Care Practice

A total of 133 physicians (52.99%) demonstrated good PCC practices. The practice scores ranged from 12 to 45 out of a possible maximum score of 45.

Regarding specific PCC practices, 30.28% of doctors reported always using family planning methods. However, only 23.9% of doctors consistently provided prenatal folic acid supplements to women with risk factors such as diabetes or epilepsy. Furthermore, 79 doctors (31.47%) had never attempted to inquire about intimate partner abuse during their consultations ( Table 5 ).

**Table 5:** Preconception care practice among physicians working at the University of Gondar Comprehensive Specialized Hospital

Items	Level	Frequency	%
**How frequent do you provide preconceptional folic acid supplementation for women with risk factor** s **like diabetes, epilepsy?**	Never Rarely Sometimes Often Always	21 39 57 74 60	8.37 15.54 22.71 29.48 23.90
**How frequent** ly **do you counsel on decreasing or stopping of alcohol consumption, cigarette smoking** , **or illicit drugs?**	Never Rarely Sometimes Often Always	7 23 62 85 74	2.79 9.16 24.70 33.86 29.48
**How frequent** ly **do you discuss about family planning before?**	Never Rarely Sometimes Often Always	3 32 107 76 33	1.20 12.75 42.63 30.28 13.15
**How frequent** ly **do you consider drug dose adjustment or discontinuation?**	Never Rarely Sometimes Often Always	4 15 67 97 68	1.59 5.98 26.69 38.65 27.09
**How frequent** ly **do you counsel on** sexually transmitted infection **screening?**	Never Rarely Sometimes Often Always	9 44 79 82 37	3.59 17.53 31.47 32.67 14.74
**How frequent** ly **do you counsel on cervical cancer screening?**	Never Rarely Sometimes Often Always	3 57 95 69 27	1.20 22.71 37.85 27.49 10.76
**How frequent** ly **do you counsel about exercise and dietary modification** ?	Never Rarely Sometimes Often Always	10 70 82 63 26	3.98 27.8 32.67 25.09 26
**How frequent** ly **do you try to assess about** i **ntimate partner violence** ?	Never Rarely Sometimes Often Always	79 79 62 25 6	31.47 31.47 24.70 9.96 2.39
**How frequent** ly **do you try to assess about mental health problem or psychosocial issues?**	Never Rarely Sometimes Often Always	54 85 66 34 12	21.51 33.86 26.29 13.55 4.78

### Bivariable and Multivariable Logistic Regression for Factors Associated with Knowledge about Preconception Care

Bivariable logistic regression identified three variables significantly associated with knowledge about PCC ( *p* < 0.2). In multivariable logistic regression, two variables remained significantly associated with PCC knowledge; current working department, physicians in certain departments had significantly higher odds of having good PCC knowledge (AOR = 3.02, 95% CI: 1.6, 5.56), ever-read PCC guidelines or protocols: physicians who had read PCC guidelines or protocols were more likely to have better knowledge (AOR = 1.82, 95% CI: 1.01, 3.2; Table 6 ).

**Table 6:** Bivariable and multivariable logistic regression for factors associated with knowledge about preconception care

Variable	Category	Knowledge about PCC		COR	CI	AOR	CI	*p* -Value
		Good	Poor					
**Current department**	Internal medicine and psychiatry gynecology and obstetrics	48 111	69 22	1 3.35	(1.8,6.0)	1 3.02	(1.6, 5.56)	0.000**
**Ever** - **read PCC guideline or protocol**	Yes No	106 73	29 42	2.28 1	(1.3,4.0)	1.82	(1.01, 3.2)	0.04*
**Perceived opinion to give PCC**	All health professionals Selected health professional	125 55	36 35	2.03 1	(1.25, 3.57)	1.12 1	(0.6, 2.0)	0.70

Abbreviations: AOR, adjusted odds ratio; CI, confidence interval; COR, crude odds ratio; PCC, preconception care.

### Bivariable and Multivariable Logistic Regression for Factors Associated with Practice of Preconception Care

Bivariable logistic regression identified three variables significantly associated with good PCC practice ( *p* < 0.2). In multivariable logistic regression, the following variables remained significantly associated with good PCC practice: physicians working in specific departments had higher odds of practicing good PCC (AOR = 2.74, 95% CI: 1.5, 4.7). Ever-read PCC guidelines or protocols: physicians who had read PCC guidelines or protocols were more likely to provide good PCC practice (AOR = 1.84, 95% CI: 1.07, 3.24). Perceived expectation of who should provide PCC: physicians who believed that all HCPs should provide PCC were more likely to practice it effectively (AOR = 2.22, 95% CI: 1.25, 3.9; Table 7 ).

**Table 7:** Bivariable and multivariable logistic regression for factors associated with the practice of preconception care

Variable	Category	Practice of PCC		COR	CI	AOR	CI	*p* -Value
		Good	Poor					
**Current department**	Internal med and psychiatry Gynecology and obstetrics	43 90	74 44	1 3.5	(2.09, 5.92)	1 2.74	(1.5, 4.7)	0.000 ^a^
**Ever** - **read PCC guideline or protocol**	Yes No	86 68	49 47	2.53	(1.5, 4.2)	1.86	(1.07, 3.24)	0.02 ^b^
**Knowledge about PCC**	Good Poor	105 28	75 43	2.15 1	(1.2, 3.7)	1.45	(0.78, 2.6)	0.236
**Perceived opinion to give PCC**	All health professionals Selected health professional	62 56	99 34	2.62 1	(1.5, 4.4)	2.21	(1.25, 3.9)	0.00 ^a^

Abbreviations: AOR, adjusted odds ratio; CI, confidence interval; COR, crude odds ratio; PCC, preconception care.

Note: ^a^ indicates strong statistical significance at the 1% level ( *p* < 0.01), and ^b^ indicates statistical significance at the 5% level ( *p* < 0.05).

Multivariate logistic regression revealed significant associations between knowledge and practice of PCC and several factors: physicians working in the obstetrics and gynecology departments were three times more likely to have good knowledge of PCC compared with those in other departments. Physicians who have read about PCC had a 1.82 times higher likelihood of possessing solid knowledge compared with those who had not read about it. Physicians who had read about PCC were also 1.86 times more likely to practice PCC well than those who had not read about it. Working in the obstetrics and gynecology department also increased the likelihood of practicing good PCC, with physicians in this department being 2.74 times more likely to provide effective care compared with those in internal medicine. Physicians who believed that all HCPs should provide PCC were 2.21 times more likely to deliver good PCC practice compared with those who thought only a few specialists should offer the service.

## Discussion

An institutional cross-sectional study was conducted to evaluate the knowledge, attitude, practice, and related factors of PCC among physicians employed at the UOGCSH.

Our survey revealed that 180 doctors (71.71%, 95% CI: 65, 77) demonstrated strong knowledge of PCC. This finding aligns with a study conducted in Addis Ababa (69.2%). 29 However, it surpasses the knowledge levels observed in studies from Hawassa by 31%, 33 Eastern Ethiopia by 60.2%, 34 Awi zone by 52%, 35 and North Wello by 49.1%. 36 This discrepancy may be attributed to the lack of PCC courses in preservice training, as well as the novelty of the PCC concept at the time of the studies. 37 Furthermore, the University of Gondar, being a renowned teaching and referral hospital, has recently incorporated PCC into the preservice curricula for various programs. The intellectual profile of the study participants may also have contributed to these higher knowledge levels.

The current finding is lower than studies conducted in Iran, where 88.3% of physicians had strong knowledge of PCC. 18 This discrepancy may be due to the fact that PCC services were available and implemented earlier in Iran, where they are considered an integral part of care and the responsibility of health care providers. This early exposure to PCC in Iran likely contributed to a higher level of provider knowledge compared with Ethiopia.

In this study, 143 physicians (56.97%, 95% CI: 50.6, 63.1) held a positive opinion about PCC. Our findings are lower than those from a study in Nigeria (79.1%), 38 but higher than a study from North Wollo (44.2%). 36 This discrepancy may be explained by the presence of more specialty programs at our teaching institution, where the inclusion of PCC services in maternity care has enhanced physician knowledge, thereby encouraging the provision of PCC. The lower result compared with the Nigerian study may be due to the fact that PCC is still in its early stages in our setting.

In our study, 133 doctors (52.9%, 95% CI: 46.6, 59.5%) demonstrated good PCC practices. This result is higher than findings from studies conducted in Nigeria (42.2%) and the West Shewa Zone (34.5%). 30 However, it is lower than studies from the South Africa (87.8%), 39 Nigeria (78.2%), 40 and Netherlands (82%), 41 and one possible explanation for this difference is that, unlike those countries, Ethiopia has not yet developed specific PCC practice guidelines; instead, PCC is generally incorporated into maternity guidelines. Additionally, the relatively new introduction of PCC services in Ethiopia may also contribute to this lower practice rate. 37

Both PCC knowledge and practice were strongly associated with the current working department, with AORs of 3.02 (95% CI: 1.6, 5.56) for knowledge and 2.74 (95% CI: 1.5, 4.7) for practice. This could be attributed to the fact that many PCC services are integrated into maternity care guidelines, which are more commonly followed by physicians working in obstetrics and gynecology departments.

Another significant factor correlated with both PCC knowledge and practice was whether or not physicians had ever-read PCC guidelines or protocols, with AORs of 1.82 (95% CI: 1.01, 3.2) for knowledge and 1.86 (95% CI: 1.07, 3.24) for practice. Physicians working in the obstetrics and gynecology departments were twice as likely to have strong PCC knowledge and practice. This finding is consistent with a study conducted in the West Shewa Zone. 30 The higher likelihood of having good PCC knowledge and practice in obstetrics and gynecology may be attributed to increased exposure to women of reproductive age, which encourages physicians to read more about PCC. Additionally, the integration of PCC services into their departments, such as interpregnancy spacing, family planning, HIV prevention, nutrition, and supplementation with iron and folic acid, may contribute to greater knowledge and more frequent practice of PCC.

The likelihood of good PCC practice was twice as high for doctors who believed that all medical practitioners should provide PCC, compared with those who believed that only a select few should offer the service. This finding aligns with studies conducted in the Oromia region 30 and Hawassa. 31 One possible explanation for this is that physicians who believe PCC should be universally provided may feel a greater sense of professional responsibility to implement it. Lastly, our study found no significant correlation between PCC knowledge and PCC practice, suggesting that knowledge alone may not be sufficient to influence the actual delivery of PCC.

## Strengths and Limitations

Primary data were used in this study, ensuring high reliability. The instrument's excellent reliability, validity, and sensitivity allowed for meaningful comparison of results using a validated tool. This study is the first of its kind in our setting and can serve as a foundation for future PCC research. However, the study had some limitations, including its single-center design due to time constraints and the potential for social desirability bias. Additionally, not all health professionals were included, which may limit the generalizability of the findings.

## Conclusion

Physicians at the UOGCSH demonstrated a strong understanding of PCC, held a positive outlook, and practiced it effectively. A good understanding and practice of PCC were significantly associated with working in the obstetrics and gynecology departments. Additionally, familiarity with PCC guidelines and protocols was found to significantly enhance PCC knowledge.

## Recommendations

To ensure physicians remain up-to-date and continue to implement evidence-based practices, it is crucial for them to prioritize reading and engaging with PCC guidelines and recommendations from various organizations. Furthermore, we recommend conducting studies that include a broader range of medical specialists, hospitals, clinics, and private health care facilities to gain a more comprehensive understanding of PCC practices across different settings. Additionally, further research should focus on exploring ways to integrate PCC services more effectively into the health care system and addressing the barriers that hinder its delivery.
